# Influence of Personal and Work Environments on Work-Life Balance Among Emergency Medical Technicians

**DOI:** 10.7759/cureus.55447

**Published:** 2024-03-03

**Authors:** Junpei Haruna, Shuji Uemura, Sachi Niiyama, Yukiko Taguchi, Saori Muranaka, Hirotoshi Inamura, Keigo Sawamoto, Hirotoshi Mizuno, Eichi Narimatsu

**Affiliations:** 1 Department of Intensive Care Medicine, School of Medicine, Sapporo Medical University, Sapporo, JPN; 2 Department of Emergency Medical Services, Department of Life Flight and Disaster Medicine, Sapporo Medical University, Sapporo, JPN; 3 Department of Emergency Medicine, School of Medicine, Sapporo Medical University, Sapporo, JPN; 4 Department of Advanced Critical Care and Emergency Center, Sapporo Medical University Hospital, Sapporo, JPN; 5 Department of Nursing, School of Health Sciences, Sapporo Medical University, Sapporo, JPN; 6 Department of Hospital Pharmacy, Sapporo Medical University Hospital, Sapporo, JPN

**Keywords:** work environments, work-life balance, emergency medical technician, first responder, emergency medicine

## Abstract

Introduction

Work-life balance (WLB) is a critical concern for emergency medical technicians (EMTs) because it significantly affects the provision of comprehensive emergency medical services (EMS). This study investigated personal and work-related factors influencing work-to-family negative spillover (WFNS), a key element of WLB, among EMTs.

Methods

A web-based survey was conducted from July 26 to September 13, 2021, among EMTs in Hokkaido, Japan. The study included 21 facilities that were randomly selected from 42 fire stations. The Japanese version of the Survey Work-Home Interaction-NijmeGen (SWING-J) was used to measure WFNS. Personal background factors, such as age, sex, years of work experience, and education, were surveyed. We also evaluated work environment factors, such as weekly working hours, monthly night shifts, monthly overtime hours, and yearly paid vacation days. Unpaired Student's t-tests, one-way analysis of variance (ANOVA), and multilevel generalized linear model (MGLM) analyses were used to explore the relationships between WFNS and personal and work-related factors.

Results

A total of 912 respondents were included in our analysis. They were predominantly male (98.2%), with an average EMT work experience of 12.7 years and a mean WFNS score of 1.16 (standard deviation (SD) = 1.67). MGLM analysis, adjusting for covariates, identified years of work experience (β = -0.129, p = 0.001), monthly overtime hours (β = 0.184, p < 0.001), and yearly paid vacation days (β = -0.170, p < 0.001) as independent factors associated with WFNS.

Conclusion

This study suggested that adjusting WFNS among EMTs could be achieved by reducing overtime hours and fostering an organized approach to paid leave within the work environment.

## Introduction

One of the primary challenges in healthcare is the notable surge in employee stress, burnout, and turnover among frontline workers in the emergency care industry. The significance of worker job satisfaction gained attention in psychology and occupational mental health around 2000, with a focus on work-life balance (WLB) as a crucial factor. Steele [1] described WLB as the simultaneous engagement of an individual with work and non-work roles, considering relationships with other social factors, such as family. Work-family conflict manifests in two directions: interference from work to family and interference from family to work [2], also known as work-to-family negative spillover (WFNS) or family-to-work negative spillover (FWNS). This conflict is influenced by individual background and work environment [3]. Studies consistently reveal a higher prevalence of WFNS compared to FWNS among healthcare professionals [4]. Research in this area supports a positive correlation between high WFNS and turnover intentions [5], highlighting the need to address these issues in the field.

Several factors influence WFNS. Studies on workplace organizations' commitment to family support within the general profession reveal that the number of family benefits, benefit utilization, and perceptions of family support from supervisors enhance WFNS [6]. In healthcare professionals, WFNS is affected by day-to-day managerial actions, responses, the impact of shift work, poorly managed rosters, and extended working hours with limited time for recovery between shifts [6].

Emergency medical services (EMS) play a crucial role in delivering pre-hospital settings worldwide [7]. While the primary responsibility of emergency medical technicians (EMTs) is providing first aid to patients with sudden illnesses, they frequently encounter traumatic and emotionally taxing situations [8], contend with dynamic and uncontrollable environments, face occupational violence, and experience physical fatigue due to irregular shift schedules [7]. Consequently, EMTs often face challenging working conditions, and maintaining appropriate WLB can be hindered by individual backgrounds and workplace factors. Despite these challenges, there are no existing reports in the literature specifically addressing WFNS in EMTs. Hence, this study aims to investigate the personal and workplace backgrounds of EMTs in Japanese fire departments as potential risk factors associated with WFNS.

## Materials and methods

This study, approved by the Ethics Committee of Sapporo Medical University, Japan, (approval no. 2-1-76), adhered to the Declaration of Helsinki and followed the Strengthening the Reporting of Observational Studies in Epidemiology (STROBE) guidelines. Informed consent was obtained from all participants prior to the survey administration. This research constitutes a secondary analysis of a 2021 survey on burnout syndrome among EMTs [9].

Between June 1-30, 2021, invitations and surveys were sent to 21 randomly selected fire departments out of 42 in Hokkaido, Japan. An introduction to the study and ethical considerations accompanied the invitations, ensuring enrollment of fire departments compliant with the study guidelines. No exclusion criteria were applied. The study committee requested background data from representatives of all invited and registered fire departments to identify the number of EMTs in each. Upon completion of the pre-survey, representatives from selected fire departments were emailed, and a facility-based cross-sectional survey of 3,215 EMTs from each fire department was conducted using ArcGIS Survey 123 (Esri, https://survey123.arcgis.com/) from July 26 to September 13, 2021. The survey questions were reviewed in advance by the co-authors (JH, SU, SN, YT, and SM).

Japanese emergency medical care system is classified into primary emergency facilities that mainly provide outpatient care, secondary emergency facilities that mainly treat severely ill patients requiring hospitalization, and emergency medical centers that treat severely ill patients who require advanced treatment. The EMS infrastructure in Hokkaido is managed by local fire departments, activated by a 119 emergency call from anywhere in Hokkaido [10]. EMTs are affiliated with municipal fire departments. In 2021, 42 fire departments and 427 ambulances were operational throughout the region [10]. Each ambulance typically has a team of three emergency personnel, including at least one emergency life-saving technician (e.g., an EMT) and a highly trained pre-hospital emergency care provider. On-site EMS personnel are responsible for choosing hospitals for patient transportation, prioritizing tertiary care facilities equipped to handle life-threatening conditions. Local medical management councils, composed of emergency physicians and specialists from various Japanese regions, play a crucial role in ensuring the quality of care provided by EMS personnel in prehospital scenarios and conducting subsequent assessments of EMS protocols [10].

Variables

Our survey comprised three components. The first addressed personal background and organizational characteristics, including age, sex, marital status, education, managerial position, full-time employment status, paramedic certification, population density of the participants’ employment area, and years of work experience. The second focused on the working environment, encompassing dispatches per year, night shifts per month, weekly work hours, monthly overtime hours, and annual paid vacations. We also assessed the frequency of EMT personnel involvement in transporting patients with COVID-19 due to potential disruptions to WLB. The third component included four items from the Japanese version of the Survey Work-Home Interaction-NijmeGen (SWINGJ) to evaluate WFNS in EMTs [11]. SWING-J, developed to assess workplace-home interactions, has demonstrated reliability and validity [12]. WFNS, defined by Alen et al. [13] as a negative load response transmitted from the workplace to the home space, was rated on a standard 4-point Likert scale (0 (mostly) to 3 (not at all)) for each SWING-J item. Total WFNS scores ranged from 0 to 24, with higher scores indicating a greater negative influence from family to work. This study compared each factor with the total WFNS score.

Bias

Simple random sampling was employed to select participants from each region's fire station, mitigating potential selection and response biases. Thus, a notable impact of selection bias on our results was not anticipated. However, certain confounding factors, including marital status, shift, and position, could still have influenced WFNS [14]. To account for this, multivariate statistics were utilized for adjustment.

Outcome

This study primarily investigated factors influencing WFNS within the WLBs of surveyed EMTs.

Statistical analysis

Descriptive statistics, including means and standard deviations (SDs) for continuous variables, and numbers with percentages for categorical variables, were calculated initially. To assess the relationship between WFNS and personal and work-related factors, unpaired Student's t-tests and one-way analysis of variance (ANOVA) were conducted for each variable. Subsequently, a multilevel generalized linear model (MGLM) analysis explored the association between high WFNS and participants' backgrounds and working environments. Covariates were introduced at the fire department level to address potential heterogeneities in managerial practices. Explanatory variables, based on prior studies and clinical perspectives, included sex, years of work experience, involvement in managing COVID-19 patients, marital status, educational status, degrees acquired, paramedic status, full-time EMT status, 24-hour shifts, number of monthly night shifts, yearly dispatch frequency, position, monthly overtime hours, and yearly paid vacations. MGLM results are presented as regression coefficients, standard errors of the mean (SEMs), β, and P values. Given the secondary nature of this analysis, the sample size was not predetermined, and statistical significance was set at P < 0.05. All analyses were performed using R 4.0.2 (R Development Core Team, Vienna, Austria) and Statistical Product and Service Solutions (SPSS, version 25; IBM Corp., Armonk, NY) statistics.

## Results

Population characteristics

The primary study encompassed 700 participants, with an additional 212 individuals included to account for missing data. This adjustment brought the total responses for calculating burnout syndrome rates to 912, resulting in a 28.4% response rate. Table 1 presents a comparison of each participant's characteristics, alongside their WFNS scores. Of the total participants, 896 (98.2%) were male, and 299 (32.8%) were in their 30s. The majority (621, 68.1%) held certification as paramedics. The EMTs, on average, had 12.7 years of work experience (SD = 8.1). The mean WFNS score was 1.16 (SD = 1.67). Notably, WFNS scores were significantly higher for EMTs with 0-5 and 6-10 years of experience compared to paramedics with > 11 years (1.36 (SD = 1.78) vs 0.85 (SD = 1.41), 1.58 (SD = 1.90) vs 0.85 (SD = 1.41)).

**Table 1:** Participant characteristics and level of WFNS Abbreviations: WFNS, Work-family negative spillover; EMT, Emergency medical technician; COVID-19, Coronavirus disease 2019 *p < 0.01

	Variables	WFNS				P value	
		n	%	mean	SD		
Total		912	100	1.16	1.67	-	
Sex							
Male		896	98.2	1.15	1.65	0.454	
Female		16	1.8	1.50	2.12		
Age category							
	20-29	293	32.1	0.97	1.49	0.097	
	30-39	299	32.8	1.23	1.75		
	40-49	258	28.3	1.33	1.76		
	50-59	59	6.5	1.00	1.61		
	60<	3	0.3	1.00	1.00		
Marital status							
Yes		691	75.8	1.2	1.7	0.069	
No		221	24.2	1.0	1.5		
Education status (bachelor’s degree)							
Yes		92	10.1	1.14	1.67	0.963	
No		820	89.9	1.16	1.67		
Position							
	Manager						
	Yes	312	34.2	1.26	1.81	0.602	
	No	600	65.8	1.11	1.59		
Full-time EMT							
Yes		276	30.3	1.15	1.71	0.593	
No		636	69.7	1.16	1.65		
Paramedics							
Yes		621	68.1	1.18	1.74	0.654	
No		291	31.9	1.11	1.50		
Population density of the participants’ employment area							
	0-5000	127	13.9	1.28	1.87	0.474	
	5001-10,000	233	25.5	1.30	1.71		
	10,001-30,000	172	18.9	1.02	1.47		
	30,001-50,000	43	4.7	1.12	1.71		
	50,001-100,000	56	6.1	1.36	1.78		
	100,001-300,000	176	19.3	1.10	1.66		
	300,001-500,000	28	3.1	078	1.17		
	500,001-	77	8.4	1.00	1.65		
Yearly dispatch frequency, n (%)							
	0-100, n (%)	501	54.9	1.17	1.67	0.972	
	101-500, n (%)	234	25.7	1.16	1.65		
	501-, n (%)	177	19.4	1.13	1.69		
Type of facility, n. (%)							
	Head office	33	3.6	1.45	1.66	0.442	
	Fire department	735	80.6	1.13	1.64		
	Field office	144	15.8	1.24	1.83		
Years of work experience							
0-5 years		191	20.9	1.36	1.78	<0.01*	0-5 years vs >11 years*
6-10 years		254	27.9	1.58	1.90		6-10 years vs >11 years*
>11 years		467	51.2	0.85	1.41		
Shift, n (%)							
	Only day shift	28	3.1	0.68	1.36	0.232	
	Double shift	199	21.8	1.07	1.60		
	Three shifts	165	18.1	1.10	1.46		
	24 hours shift	520	57.0	1.24	1.77		
Monthly night shifts							
0-10 times		98	10.7	1.08	1.95	0.622	
>11 times		813	89.1	1.17	1.63		
Weekly work hours							
0-48 hours		493	54.1	1.08	1.52	0.099	
>49 hours		419	45.9	1.26	1.82		
Monthly overtime hours							
<5 hours		714	78.3	1.15	1.67	0.587	
6-10 hours		91	10.0	1.31	1.74		
>11 hours		107	11.7	1.07	1.62		
Yearly paid vacations							
<10 days		369	40.5	1.13	1.65	0.393	
10-20 days		335	36.7	1.25	1.73		
>21 days		208	22.8	1.06	1.60		
Involved in COVID-19 patient management							
Yes		787	86.3	1.18	1.70	0.391	
No		125	13.7	1.04	1.44		

Factors associated with WFNS in EMTs

**Figure 1:**
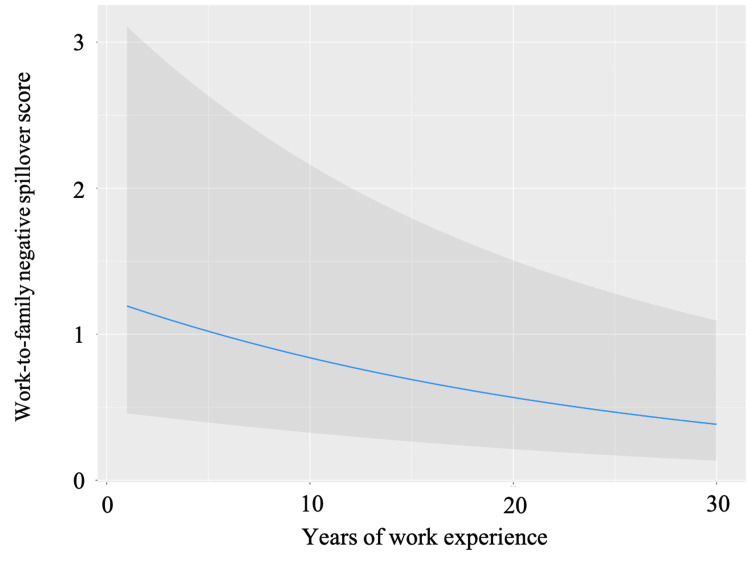
The association between years of work experience and WFNS scores The association between years of work experience and WFNS scores after adjusting for pre-defined covariates. The gray area indicates a 95% confidential interval. Abbreviation: WFNS, Work-family negative spillover

**Figure 2:**
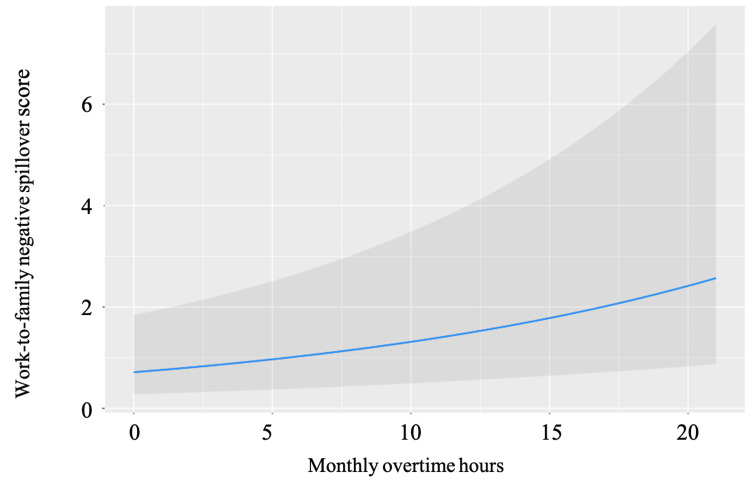
The association between monthly overtime hours and WFNS scores The association between overtime hours per week and WFNS scores after adjusting for pre-defined covariates. The gray area indicates a 95% confidential interval. Abbreviation: WFNS, Work-family negative spillover

**Figure 3:**
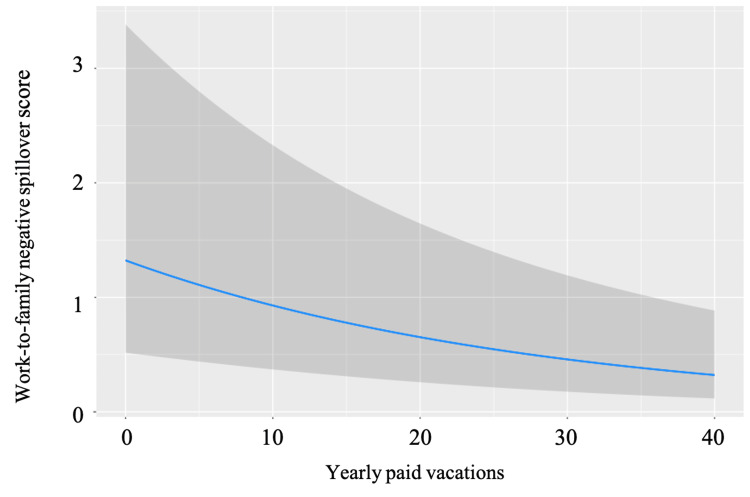
The association between yearly paid vacations and WFNS scores The association between yearly paid vacations and WFNS scores after adjusting for pre-defined covariates. The gray area indicates a 95% confidential interval. Abbreviation: WFNS, Work-family negative spillover

The MGLM analysis results, adjusted for predefined covariates, are outlined in Table 2. Significant associations with WFNS scores were identified for the following factors: years of work experience (β = -0.129, p = 0.001; Figure 1), monthly overtime hours (β = 0.184, p < 0.001; Figure 2), and yearly paid vacations (β = -0.170, p < 0.001; Figure 3).

**Table 2:** Multiple regression analysis with WFNS as the dependent variable Abbreviations: WFNS, Work-family negative spillover; β, standardized coefficient; B, partial regression coefficient; R2, coefficient of determination; SE B, standard error of partial regression coefficient; EMTs, emergency medical technicians; COVID-19, coronavirus disease 2019

Variables	B	SE B	β	P-value
Sex (male)	-0.374	0.405	-0.029	0.357
Marital status	0.247	0.131	0.063	0.059
Education’ status, degrees	-0.170	0.180	-0.031	0.346
Years of work experience	-0.027	0.008	-0.129	0.001
Paramedics	-0.031	0.115	-0.009	0.788
Full-time EMT	0.046	0.145	0.013	0.752
Position, manager	0.247	0.134	0.070	0.065
Yearly dispatch frequency				
0-100	-	-	-	-
101-500	-0.030	0.137	-0.008	0.826
>501	-0.050	0.177	-0.012	0.777
24 hours shift	0.131	0.108	0.039	0.227
Monthly night shifts	0.010	0.024	0.013	0.692
Weekly hours worked	0.000	0.003	0.002	0.945
Monthly overtime hours	-0.058	0.011	0.184	<0.001
Yearly paid vacations	-0.031	0.006	-0.170	<0.001
Involved in COVID-19 patient management	-0.025	0.051	-0.016	0.620
R^2^	0.362			<0.001
Adjusted R^2^	0.331			

## Discussion

This study explored risk factors for elevated WFNS in EMTs. Results indicated that fewer years of work experience was associated with higher WFNS. Additionally, increased monthly overtime hours and fewer annual paid vacations were associated with elevated WFNS. Our findings underscore the need for targeted interventions, emphasizing organizational support - such as emotional assistance and technical education - for EMTs with limited work experience. Emotional assistance includes helping EMTs seek support from others and encouraging them to express their feelings and work together to find solutions during times of emotional instability. Moreover, addressing broader work environment issues, such as managing overtime hours and ensuring sufficient paid vacation, is crucial for all EMTs. Previous research on WFNS reported mean scores of 16.1 in Romanian workers [15], 7.36 in Japanese dual-earner couples [16], and 2.6 in Spanish teachers and researchers [17]. Similarly, Japanese nurses reported a mean score of 7.8 [18]. While WFNS scores varied across countries and professions, the EMTs in our study generally exhibited lower scores compared to previous reports. However, a subset of EMTs with notably high WFNS scores was identified.

The primary finding of this study indicates that EMTs with fewer years of experience encounter more WFNS effects. Previous research in healthcare fields other than EMT has linked years of experience with WLB. Physicians and nurses with fewer years of experience face increased work stress, adversely affecting family life [19]. Conversely, more years of experience are associated with improved WLB among EMTs [20]. Reports also suggest that paramedics with fewer years of experience exhibit a higher burnout prevalence [21]. This could be due to their lack of skills and knowledge to handle high-stress situations, leading to feelings of inadequacy and anxiety [22]. Addressing these issues may involve organizational support, such as psychological and educational assistance for less experienced EMTs.

The second significant finding highlights that longer monthly overtime hours lead to heightened WFNS effects. A survey on overtime hours of EMTs revealed that extended shifts result in mental, physical, and emotional exhaustion [23]. Doubling or tripling work hours amplifies negative effects in both short- and long-term contexts, increasing burnout and cynicism risks [23]. Factors such as daily work hours and overtime have also been linked to mental health issues, including post-traumatic stress disorder, depression, and anxiety [14]. Consequently, increased overtime hours contribute to burnout and mental health concerns in EMTs, negatively impacting WLB.

A third key discovery underscores that fewer annual paid vacation days correlate with a higher degree of WFNS effect. Paid leave is a critical WLB policy that organizations can implement to assist employees in managing work and non-work demands [24]. The International Labour Organization notes that the availability of various forms of paid leave influences WLB [25]. However, the availability and utilization of paid leave may vary based on sector, country, organizational culture, and individual employee preferences [26]. EMTs and healthcare professionals, facing challenges such as high work pressure, long hours, emotional exhaustion, and burnout, can benefit from paid leave [27]. It helps reduce work-related stress, enhance well-being, and improve overall quality of life [28].

Finally, our findings may have been influenced by the COVID-19 pandemic. While the survey coincided with the peak of the pandemic in Japan [29], it is imperative to interpret the results mindful of varied pandemic impacts across countries and the potential influence of survey timing on responses.

Implication for clinical practice

Improving WFNS is commonly achieved through strategies such as family leave, flexible hours, child care assistance (e.g., subsidized or on-site care), compressed work weeks, telecommuting, and job sharing [5]. These tactics are prevalent in modern organizations, forming a key part of their approach to attracting, motivating, and retaining essential personnel [30]. The discussion suggests that enhancing working conditions, such as favorable hours and family leave, could enhance the care provided by front-line EMTs involved in pre-hospital emergency care. However, since most EMTs work in shifts, these benefits may be challenging to implement. Offering alternative work arrangements and schedules to EMTs could be a viable solution, allowing employees to balance their professional and family commitments better. Addressing individual needs within the EMT workforce may contribute to an overall improvement in WFNS, potentially reducing obstacles to enhancing pre-hospital emergency care.

Strengths and limitations

To our knowledge, this marks the inaugural examination of factors influencing WFNS in EMTs. However, we recognize several pivotal limitations. Firstly, the study focused exclusively on EMTs in a single Japanese province, relying on a constrained survey sample, potentially introducing notable selection bias. Despite this, we mitigated the bias through adjustments in our multivariate analysis, encompassing various covariates for WFNS. Nevertheless, replication in a more diverse, multinational cohort is essential for robust comparison and broader generalization. Secondly, we could not analyze the backgrounds of non-respondent EMTs. With a web-based survey response rate of approximately 30%, it is plausible that some non-participants harbored strong views on WFNS. Thirdly, most of the subjects in this study were male. Since the Japanese Fire Administration reports that approximately 90% of EMTs are male, it is possible that the male-to-female ratio in this survey was also heavily skewed toward males [10]. Future studies with larger samples are needed because WLB may vary by sex.

## Conclusions

The study revealed that lower work experience, increased monthly overtime hours, and fewer annual paid vacations were independently associated with higher WFNS in EMTs. To mitigate WFNS, we underscore the need for targeted interventions, such as field technical education, for EMTs with limited work experience. In addition, work practices should be customized to meet the individual needs of EMTs.
